# Xiao Yao San Improves Depressive-Like Behavior in Rats through Modulation of *β*-Arrestin 2-Mediated Pathways in Hippocampus

**DOI:** 10.1155/2014/902516

**Published:** 2014-07-07

**Authors:** Xiaoxia Zhu, Oudong Xia, Weili Han, Meng Shao, Linlin Jing, Qin Fan, Yuanliang Liu, Jianxin Diao, Zhiping Lv, Xuegang Sun

**Affiliations:** ^1^Department of Traditional Chinese Medicine, Nanfang Hospital, Southern Medical University, No. 1838, Guangzhou 510515, China; ^2^The Key Laboratory of Molecular Biology, State Administration of Traditional Chinese Medicine, School of Traditional Chinese Medicine, Southern Medical University, Guangzhou, Guangdong 510515, China; ^3^Dean's Office, Zhujiang Hospital, Southern Medical University, Guangzhou 510282, China; ^4^Hygiene Detection Center, School of Public Health and Tropical Medicine, Southern Medical University, Guangzhou 510515, China; ^5^Traditional Chinese Medicine Integrated Hospital, Southern Medical University, Guangzhou, Guangdong 510305, China

## Abstract

Xiao Yao San (XYS) is a classical Chinese medicine formula that has been widely used to treat mood disorders for hundreds of years. To confirm the effect of XYS and better understand its underlying mechanism, high-performance liquid chromatography-mass spectrometry analysis-based quality control of XYS extracts and proteomics-based identification of differential proteins in the hippocampus were adopted in social isolation and chronic unpredictable mild stress- (CUMS-) treated rats. The depressive-like behavior of rats induced by CUMS resembled the manifestation of human depression. The upregulated corticosterone (CORT) and urocortin 2 (UCN2) levels demonstrated the existence of hypothalamic-pituitary-adrenal (HPA) axis hyperactivity. XYS was effective in ameliorating the depressive-like behavior and downregulating UCN2 and CORT. XYS decreased the expression of serine/threonine-protein phosphatase 2A subunit B and increased the expression of *β*-arrestin 2. The expressions of brain-derived neurotrophic factor (BDNF), tyrosine receptor kinase B (TrkB), and mammalian target of rapamycin (mTOR) were also elevated by XYS. In conclusion, XYS improves social isolation and CUMS-induced depressive-like behavior and ameliorates HPA hyperactivation through the downregulation of corticotrophin releasing hormone (CRH) receptor 2. The upregulation of BDNF/TrkB and the phosphorylation of mTOR require *β*-arrestin 2 as a scaffold to regulate stress signaling.

## 1. Introduction

Major depressive disorder (MDD) is a mental disorder characterized by episodes of all-encompassing low mood accompanied by low self-esteem and loss of interest or pleasure in normally enjoyable activities. The prevalence of 12-month and lifetime DSM-IV MDD among adults in the United States is 5.28% and 13.23%, respectively [1]. MDD ranks second among the diseases with the largest number of years lived with disability and fifth in the disability-adjusted life-year ranking of the top 30 diseases and injuries in 2010 [2]. Longitudinal studies confirm a rising prevalence of MDD, suggesting that we are indeed in the midst of an MDD epidemic [3]. Furthermore, almost half of MDD patients think about suicide or want to die. Therefore, MDD uses many social and health care services and continues to be a serious personal and public health problem.

Serotonin deficiency is the prevailing hypothesis of MDD [4]. Selective serotonin reuptake inhibitors account for about 60% to 80% of the market share of antidepressants [5]. However, 5-hydroxytryptamine (5-HT) self-depletion does not cause depression in healthy volunteers and does not worsen symptoms in unmedicated depressed patients [6]. Hypothalamic-pituitary-adrenocortical (HPA) hyperactivity is a result of the deficit negative feedback regulation of the axis. HPA is causally linked to the development of depressive symptoms and occurs in 30% to 50% of acutely depressed patients [7]. The hippocampus is responsible for the learning and cognition part of the depressive disorder [8]. Neurotrophin deficiency in the hippocampus [9] is involved in the pathogenesis of MDD. However, these various hypotheses are a partial understanding of MDD and are far from being mutually exclusive [10].

Many traditional Chinese herbal formulas have been reported to have antidepressant-like effects [11–14]. Among them, Xiao Yao San (XYS) is the most commonly used formula in treatment of depressive mood disorders for it has been widely prescribed for about nine hundred years from the Song dynasty. MDD patients usually manifest liver stagnation and spleen deficiency according to theory of traditional Chinese medicine. XYS can soothe the liver, invigorate the spleen, and nourish the blood so it has been widely used to treat MDD. Moreover, XYS upregulates the 5-HT content [15], downregulates corticotropin-releasing hormone (CRH) expression in the hypothalamus [16], and increases the expression of brain-derived neurotrophic factor (BDNF) in the hippocampus [17]. However, the mechanism of XYS and the pathophysiology of MDD remain ambiguous. A proteomics-based approach was used to identify the differentially expressed hippocampal protein profiles of XYS in a depressive model induced by social isolation and chronic unpredictable mild stress (CUMS). This study aimed to establish a more integrative framework for MDD by unifying HPA hyperactivity and hippocampal neuron deficiency and to provide a deeper understanding of the mechanism of XYS.

## 2. Materials and Methods

### 2.1. Preparation of Drugs

XYS is composed of Bupleuri Radix (root of* Bupleurum *Chinese DC), Angelicae sinensis Radix (root of* Angelica sinensis* (Oliv.) Diels), Paeoniae Radix Alba (root of* Paeonia lactiflora* Pall.), Atractylodis Macrocephalae Rhizoma (root and rhizome of* Atractylodes macrocephala* Koidz), Poria (fungus nucleus of* Poria cocos* (Schw.) Wolf), Zingiberis Rhizoma Recens (root and rhizome of* Zingiber officinale* Rosc.), Menthae Haplocalycis Herba (overground parts of* Mentha haplocalyx* Briq.), and Glycyrrhizae Radix et Rhizoma (root and rhizome of* Glycyrrhiza uralensis* Fish.). The raw herbs for XYS were purchased from the Affiliated Nan Fang Hospital of Southern Medical University. The herb materials were authenticated by Professor Liu Q, an expert on pharmacognostical identification in School of Traditional Chinese Medicine, Southern Medical University. The voucher specimens were deposited in the storage cabinet of Chinese traditional medicine of School of Traditional Chinese Medicine, Southern Medical University. A total of 185 g of herbs was mixed at a ratio of 6 : 6 : 6 : 6 : 6 : 2 : 2 : 3 (dry weight). Aqueous extracts of XYS were extracted at 80°C by stirring for 1 h using 10 volumes of distilled water (v/m). The extracts were centrifuged at 1,500 ×g at room temperature. The supernatant was collected and subjected to condensation under reduced pressure at 70°C to obtain the semisolid XYS solution [18]. Quality of XYS was confirmed by high-performance liquid chromatography-mass spectrometry analysis (see Figure S1 in Supplementary Materials available online at http://dx.doi.org/10.1155/2014/902516). XYS was suspended again in distilled water at a final concentration of 1.9 g/mL. Fluoxetine hydrochloride (Lilly Suzhou Pharmaceutical Co., LTD, number J20080016) was purchased from the Affiliated Nan Fang Hospital of Southern Medical University and dissolved in distilled water to a final concentration of 0.2 mg/mL. The solution was stored in aliquots at −20°C.

### 2.2. Animals and Experimental Procedures

All procedures involving laboratory animals were in accordance with the guidelines of the Instituted Animal Care and Use Committee of Southern Medical University. All protocols were submitted and validated by the Animal Care Ethics Committee of Southern Medical University (number 2012-065). A total of 40 male Sprague-Dawley rats, weighing 200 ± 20 g, were purchased from the Center of Experimental Animals, Southern Medical University. The animals were maintained under controlled conditions (22°C, 12 h/12 h dark/light cycle) in a conventional animal colony for one week to adapt to the new environment.

Rats were assigned randomly into four groups: control, model, XYS, and fluoxetine. Five animals per cage in the control group were housed and allowed free access to food and water. Animals in the other three groups underwent social isolation and CUMS procedures (Table  S1). Briefly, each animal was socially isolated by placing each animal in a separate cage and underwent CUMS protocol. CUMS procedure involves a variety of mild stressors: (1) food deprivation for 24 h, (2) water deprivation for 24 h, (3) exposure to an empty bottle for 1 h, (4) cage tilt (45°) for 7 h, (5) overnight illumination, (6) soiled cage (200 mL of water in 100 g of sawdust bedding) for 24 h, (7) forced swimming at 8°C for 30 min, (8) physical restraint for 3 h, and (9) exposure to a foreign object (e.g., a piece of plastic) for 24 h [19]. About 19 g/kg/d XYS, 2 mg/kg/d fluoxetine, and an equivalent volume of distilled water (for model and control groups) were administrated by gavage using a tube twice a day.

### 2.3. Behavior Tests

The open-field apparatus was a four-sided 80 × 80 × 40 cm wooden enclosure, with the side walls painted black and the floor painted khaki divided into 25 equal squares by black lines. The open-field tests were performed in a dimly lit (25 W) and quiet (less than 60 dB) room. Each rat was gently placed at the center of the square and observed for consecutive 5 min periods. All animals were tested once between 9 am and 1 pm. The apparatus was wiped with 70% ethanol and dried between rats. For scoring, a video-tracking system (EthoVision, Noldus, Wageningen, Holland) was used to record the grids travelled and the number of rears [20].

Sucrose preference test was performed after rats were provided with a free choice between two bottles (one with 1% sucrose solution and another with tap water) for 24 h. The position of the bottles was switched after 12 h to prevent the possible effects of side preference in drinking behavior. No previous food or water deprivation was applied before the test. The consumption of water and sucrose solution was estimated simultaneously in control and experimental groups by weighing the bottles. The sucrose intake was calculated as an amount of consumed sucrose in grams. The preference for sucrose was calculated as the percentage of the consumed sucrose solution of the total amount of liquid drunk [21].

Food consumption evaluations were made after fasting for 24 h. This further evaluation was necessary because food deprivation was used in our behavioral tasks as a mild stressor [22].

Body weight was measured and recorded on the last day of the week.

The rats were sacrificed after the behavioral tests. The whole brains of three rats in each group were removed after accepting heart perfusion. The brains were fixed in 4% paraformaldehyde solution for further histopathological assessment and immunohistochemistry. The left hippoacmpi of the remaining rats were flash-frozen in liquid nitrogen and stored at −80°C for protein analysis. The right hippocampi were kept in 10 volumes of RNAlater Solution (Ambion, Life technologies, Carlsbad, CA, USA) for polymerase chain reaction (PCR) analysis.

### 2.4. Hormone Measurement

Blood was collected from the abdominal aorta by a puncturing needle. Cerebrospinal fluid (CSF) was extracted from the foramen magnum by a 1 mL syringe. Blood samples were placed at room temperature for 30 min before centrifugation for 10 min at 3,000 ×g.

Serum adrenocorticotropic hormone (ACTH) and corticosterone (CORT) were analyzed using an IMMULITE 2000 immunoassay system with ACTH and CORT immunoassay kits.

The serum and the CSF levels of CRH and urocortin-2 (UCN2) were detected by a microtiter plate reader (Victor3_V_, Perkin Elmer, Waltham, MA, USA) with rat CRH enzyme-linked immunosorbent assay (ELISA) kit (Baoman Biotech, Shanghai) and rat UCN2 ELISA kit (Chang Yi Chemical, Chemical, Shanghai).

### 2.5. Histopathological Assessment and Nissl's Staining

In the histopathological examination, paraformaldehyde solution was used to fix the paraffin-embedded brain tissues that were cut into serial sections (3 *μ*m). The slices were stained with hematoxylin and eosin and Nissl's solution in a routine procedure.

### 2.6. Two-Dimensional Electrophoresis (2D) and Protein Identification

The 2D electrophoresis was performed as previously reported and repeated for three times [18]. Briefly, hippocampal samples containing 300 *μ*g of protein were loaded per tube in an isoelectric focusing system (IPGphor II, GE). The samples were isoelectrofocused and separated with sodium dodecyl sulfate-polyacrylamide gel (SDS-PAGE). The gels were silver-stained. The protein spots that either increased or decreased for more than twofold were selected for matrix-assisted laser desorption/ionization time of flight mass spectrometry (MALDI-TOF MS) identification. The mascot software package and the database of SwissProt were used to match the mass of peptides [23].

### 2.7. Western Blot Analysis

Western blot analysis was performed as previously described [24]. Briefly, the protein lysates were loaded onto 10% SDS-PAGE for separation, electrotransferred onto PVDF membranes, and blocked in 5% nonfat milk in Tris-buffered saline Tween. The membranes were incubated overnight with primary antibodies, such as anti-*β*-arrestin 2 (Cell Signaling Technology, CST, clone C16D9, 1 : 750 dilution), anti-extracellular signal-regulated kinase (ERK, CST, Clone 137F5, 1 : 800 dilution), anti-phospho-ERK (Thr202/Tyr204, CST, 1 : 800 dilution), anti-tyrosine receptor kinase B (TrkB, Bioss, clone bs-0175R, 1 : 400 dilution), anti-BDNF (epitomics, 1 : 4000 dilution), anti-serine/threonine-protein phosphatase 2A subunit B (PP2A b, Abcam, 1 : 800 dilution), anti-PP2A c (Abcam, 1 : 750 dilution), anti-CRH receptor 1 (CRHR1, Bioss, 1 : 400 dilution), anti-CRH receptor 2 (CRHR2, Abcam, 1 : 800 dilution), anti-mammalian target of rapamycin (mTOR, CST, 1 : 800 dilution), anti-phospho-mTOR (phospho-S2448, Abcam, 1 : 800 dilution), and anti-*β*-actin (Clone TA-09, ZSGB-BIO, 1 : 1000) at 4°C. This procedure was followed by incubation with horseradish peroxidase- (HRP-) conjugated secondary antibody. The results were visualized with enhanced chemiluminescence (GE Healthcare Bio-science, Uppsata, Sweden). Images were captured and documented with a CCD system (Imagestation 2000MM, Kodak, Rochester, Rochester, NY, USA). The quantitative analysis of these images was performed using Molecular Imaging Software Version 4.0 (provided by Kodak 2000MM System). The optical density was normalized against that of the *β*-actin.

### 2.8. Immunohistochemistry

The paraffin-embedded hippocampal sections were deparaffinized, rehydrated, and pretreated with hydrogen peroxidase in phosphate buffer solution. Heat-induced antigen retrieval was conducted. After blocking with the appropriate antisera, sections were incubated with anti-*β*-arrestin 2 (CST, 1 : 50 dilution), anti-BDNF (epitomics, 1 : 100 dilution), anti-TrkB (Bioss, 1 : 100 dilution), anti-phospho-ERK (Thr202/Tyr204, CST, 1 : 50 dilution), anti-CRHR1 (Bioss, 1 : 100 dilution), and anti-CRHR2 (Abcam, 1 : 50 dilution). After incubation with HRP-conjugated secondary antibody and tyramide amplification followed by streptavidin-HRP, positive signals were visualized by a diaminobenzidine kit and counter-stained with hematoxylin.

### 2.9. Quantitative RT-PCR Detection

RNA isolation and quantitative real-time PCR (qRT-PCR) were performed as described previously [25]. Briefly, total RNA from each indicated group was extracted using Trizol reagent. Complementary DNA was synthesized, and qRT-PCR was performed on a Stratagene Mx3005P QPCR System (La Jolla, CA, USA). The sequences of primers are listed in Table  S2. PCR results were analyzed using Opticon Monitor Analysis 2.0 software (Bio-Rad Laboratories, Hercules, CA, USA). Relative mRNA expression was quantified by subtracting the glyceraldehyde 3-phosphate dehydrogenase threshold cycle (*C*_*t*_) value from the *C*_*t*_ value of the genes of interest. It is expressed as 2^−ΔΔ*C*_*t*_^.

### 2.10. Statistical Analysis

All data were expressed as mean ± SD and analyzed using an SPSS statistical package (version 13.0, Armonk, NY, USA). Mean values were compared through one-way ANOVA. A multicomparison was also conducted. Data were tested through homogeneity test for variance. The mean values of the homogenous variances were compared using ANOVA. The differences between the two groups were analyzed based on the test of least significance difference. The mean values of the nonhomogeneous variances were compared using Welch's test. The differences between the two groups were analyzed using the Games-Howell test. *P* < 0.05 was considered statistically significant.

## 3. Results

### 3.1. Effect of XYS on Behavioral Changes in Socially Isolated and CUMS-Induced Depressive Rats

All rats were active, were brisk, and were in good mood fitness with white and lustrous furs before the experiment. In the beginning, the rats were agitated when restrained and frequently groomed after being unclenched. Repeated struggling was observed when the rats were forced to swim. After 10 d, the rats in the model group became numb and showed unnatural passivity manifested as being lazy and sluggish and dodging the nooks. At the end of the 21 d of CUMS, the rats showed various depressive behaviors, such as poor appetite, droopy whiskers, low-pitched voice, lusterless fur, loose stool, and slow responsiveness. XYS treatment ameliorated the depressive behavior by improving the appetite and responsiveness of the rats.

**Figure 1:**
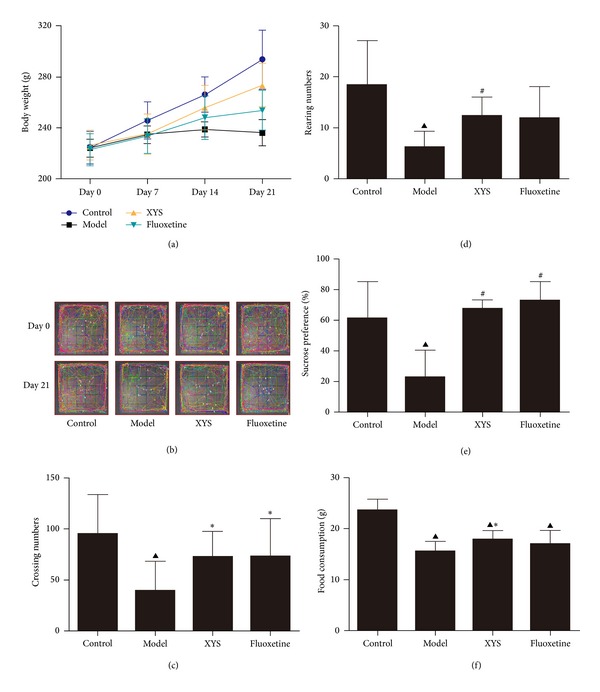
Effects of XYS on body weight and behavior of rats treated with social isolation and CUMS. Body weight was measured once a week (a). A battery of behavioral tests was initiated 21 d after modeling, and the following parameters were measured: crossing trajectories (b), crossing numbers (c), rearing numbers (d), sucrose preference (e), and food consumption (f). Data are expressed as mean ± SD, *n* = 10 per group. ^△^ *P* < 0.05, ^▲^ *P* < 0.01 versus control, *P < 0.05, ^#^ *P* < 0.01 versus model.

Social isolation and CUMS treatment decreased the body weight of rats at 14 d and 21 d (*P* < 0.01). Moreover, XYS and fluoxetine significantly improved the body weight loss at 14 d and 21 d (*P* < 0.01) (Figure 1(a)).

At 21 d, the number of grids crossed with all paws, and the number of rearing responses significantly decreased in the model group unlike in the control (*P* < 0.01). XYS had no significant effects on rats without social isolation and CUMS treatment (Figure  S2). XYS and fluoxetine significantly increased the number of crossed grids (*P* < 0.05), and XYS increased the rearing responses (*P* < 0.01) (Figures 1(b)–1(d)).

Social isolation and CUMS significantly decreased the percentage of sucrose consumption in the model group unlike the control group (*P* < 0.01). Long-term treatment with XYS and fluoxetine significantly increased the percentage of sucrose consumption in socially isolated and CUMS-exposed rats (*P* < 0.01) unlike the model group (Figure 1(e)). Moreover, food consumption significantly decreased unlike the control group (*P* < 0.01). XYS significantly increased food consumption (*P* < 0.05), whereas fluoxetine failed to increase food consumption unlike the model group (Figure 1(f)).

### 3.2. Effect of XYS on ACTH, CORT, CRH, and UCN2 in Socially Isolated and CUMS-Induced Depressive Rats

**Figure 2:**
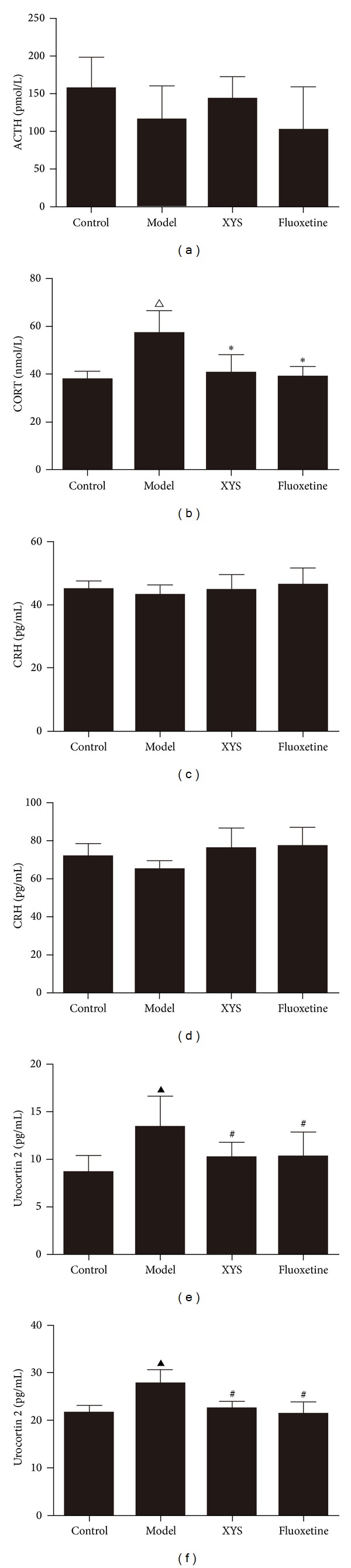
Effects of XYS on serum and cerebrospinal fluid hormone levels in depressive rats. Effect of XYS on serum ACTH (a), serum CORT (b), serum CRH (c), CSF CRH (d), serum urocortin 2 (e), and CSF urocortin (f). Data are expressed as mean ± SD, *n* = 6 per group. ACTH: adrenocorticotropic hormone; CORT: corticosterone; CRH: corticotropin-releasing hormone; CSF: cerebrospinal fluid. ^△^ *P* < 0.05, ^▲^ *P* < 0.01 versus control, *P < 0.05, ^#^ *P* < 0.01 versus model.

The serum CORT significantly increased in the model group unlike the control group (*P* < 0.05); serum and CSF UCN2 also increased (*P* < 0.01). XYS and fluoxetine significantly decreased the CORT level (*P* < 0.05) as well as the serum and CSF UCN2 levels (*P* < 0.01). No significant difference was observed in the serum and CSF levels of CRH and serum ACTH based on the ANOVA (Figure 2).

### 3.3. Effect of XYS on Morphology and Neuron Number in the Hippocampus

**Figure 3:**
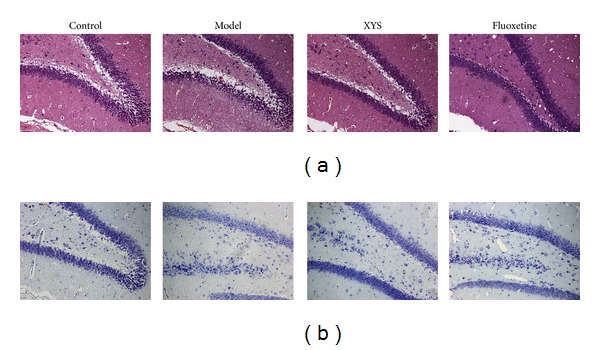
Effect of XYS on the histologic structure of dentate gyrus (DG). HE staining (a) and Nissl's staining of the DG hippocampus (b).

HE staining showed that neuronal cells in the hippocampus were neatly arranged in the control group (Figure 3(a)). Neuronal cell bodies and basophilic granules decreased in the model group. The neuronal cell numbers improved, and Nissl's body was increased by XYS (Figure 3(b)).

### 3.4. Effects of XYS on the Expressions of CRHR1 and CRHR2

**Figure 4:**
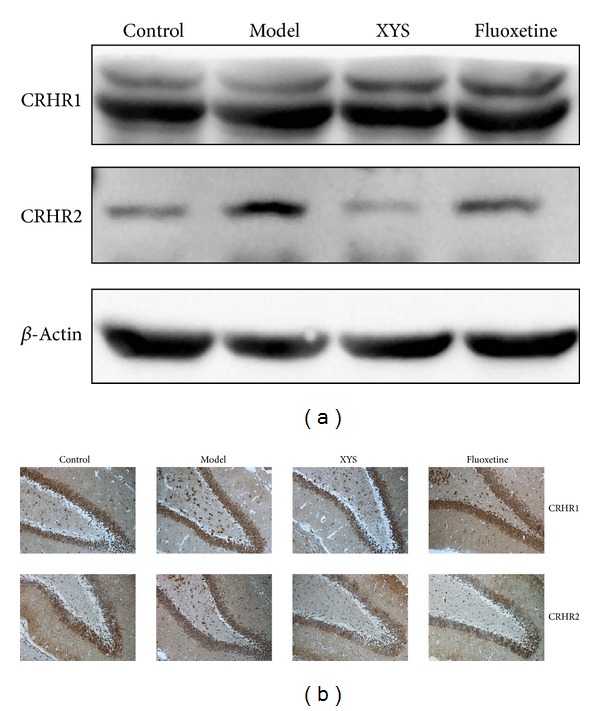
Representative Western blot analysis (a) and immunohistochemical staining (b) of CRHR1 and CRHR2 in the hippocampus. CRHR: corticotropin-releasing hormone receptor. Refer to Table 2 for the semiquantitative analysis of the above images.

The expression of CRHR2 was upregulated in the model group unlike the control group. The expression was significantly downregulated by XYS and fluoxetine treatment. No significant difference was observed in the expression of CRHR1 among the four groups (Figure 4).

### 3.5. Proteomics Analysis of Differentially Expressed Phosphorylated Proteins

**Figure 5:**
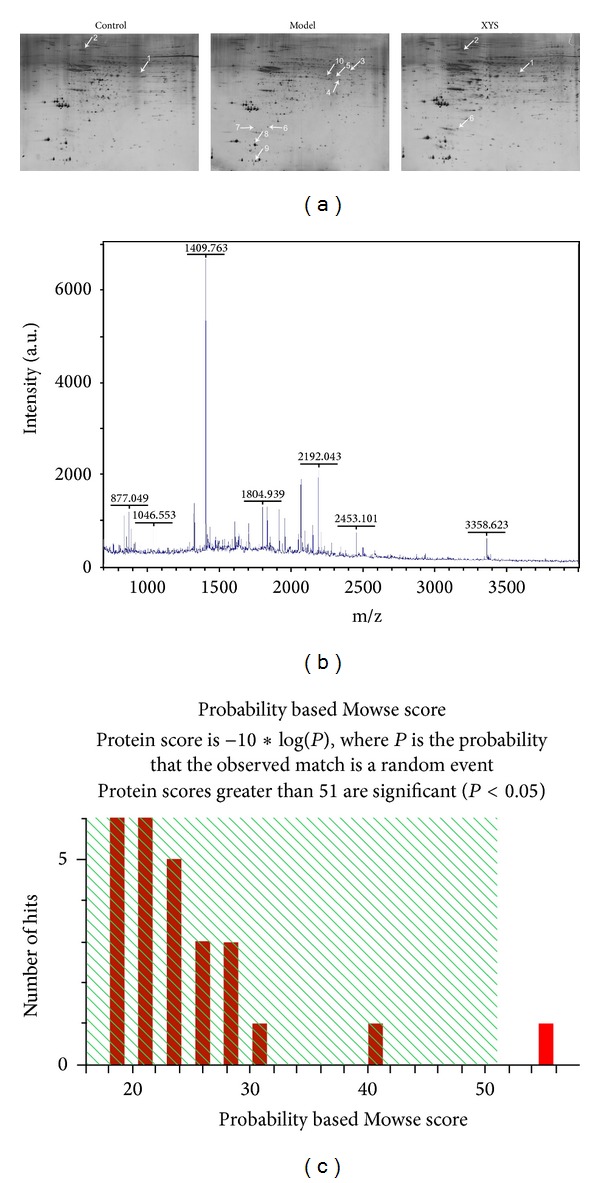
2D gel images of protein expression and MALDI-TOF MS identification of samples from the hippocampus. 2D gel image of protein expression (a). Mascot search results of spot 7 (b). MALDI-TOF MS was obtained from spot 7 after trypsin digestion (c). 2DE, 2D electrophoresis, MALDI-TOF MS, and matrix-assisted laser desorption/ionization time of flight mass spectrometry.

Based on the reproducible 2D gel electrophoresis, two downregulated and eight upregulated spots were observed in the model group unlike the control group (Figure 5(a)). The two upregulated spots by XYS were identified as prohibitin and proteasome subunit beta type-6. Among the eight spots downregulated by XYS, five spots were identified as tubulin alpha-1A chain, DCN1-like protein 3, serine/threonine-protein phosphatase 2A 55 kDa regulatory subunit B alpha isoform (PP2Ab), tubulin beta-2C chain, sodium channel, and clathrin linker 1 (Table 1).  Figure 5(b) illustrates the peptide mass fingerprint obtained from the seven spots.  Figure 5(c) shows the mascot research results.

### 3.6. Verification of XYS on the Expression of PP2Ab and on the Phosphorylation of Akt and mTOR

**Figure 6:**
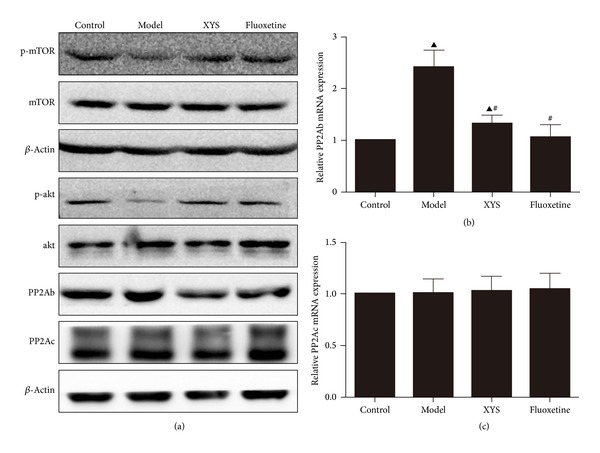
Representative images of the expressions of phospho-mTOR, phospho-Akt, PP2Ab, and PP2Ac in the hippocampus (a), PP2Ab (b), and PP2Ac (c). mRNA expression levels of the hippocampus by qPCR. p-mTOR: phospho-mammalian target of rapamycin; PP2A: serine/threonine-protein phosphatase 2A. Refer to Table 2 for the semiquantitative analysis of the above images.

The expression of PP2Ab was increased by social isolation and CUMS treatment; the expression of PP2Ac was not affected. Quantitative RT-PCR also showed that PP2Ab, not PP2Ac, was downregulated by XYS. The phosphorylation of mTOR and protein kinase B (Akt) was significantly upregulated by XYS (Figure 6).

### 3.7. Effect of XYS on the Expressions of *β*-Arrestin 2 and BDNF and on the Phosphorylation of ERK

**Figure 7:**
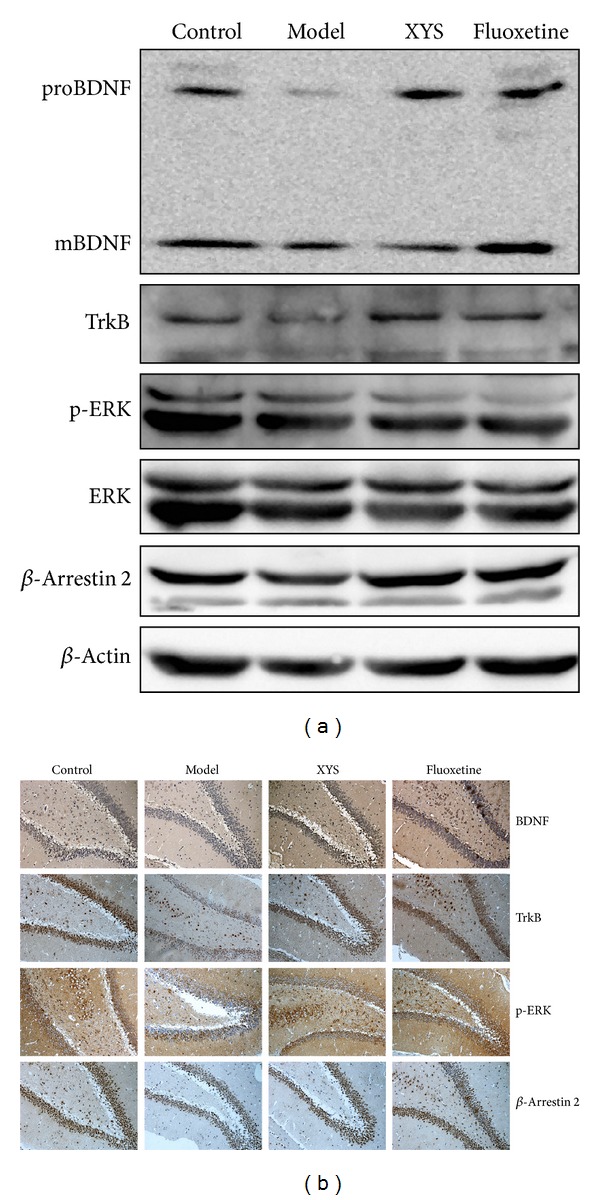
Representative Western blot analysis (a) and immunohistochemical staining (b) of BDNF, TrkB, p-ERK, ERK, and *β*-arrestin 2 in the hippocampus. BDNF: brain-derived neurotrophic factor; TrkB: tyrosine kinase receptor B; ERK: extracellular signal-regulated kinase. Refer to Table 2 for the semiquantitative analysis of the above images.

The decreased expression of *β*-arrestin 2, BDNF, TrkB and the phosphorylation of ERK was observed in the model group unlike the control group. XYS increased the expression of *β*-arrestin 2, BDNF, and TrkB and the phosphorylation of ERK. The expression patterns of BDNF, TrkB, and *β*-arrestin 2 and the phosphorylation of ERK were further confirmed by immunohistochemistry (Figure 7).

## 4. Discussion

An animal model of CUMS-induced depression was originally established by Katz [26] and modified by Willner [27] to simulate the pathogenesis of depression in humans. The CUMS paradigm causes anhedonia, which is the loss of interest in normally pleasurable and rewarding activities. The use of the model is a well-validated method to cause depression, and the model has face and predictive validity [27]. Social isolation aggression can potentiate anxiety and depressive-like behavior in isolated mice subjected to unpredictable chronic mild stress [28]. Therefore, social isolation combined with CUMS is adopted to induce the depressive behavior in rats. Poor appetite, droopy whiskers, slow responsiveness of rats, elevated CORT and UCN 2, open field changes, and anhedonia deficiency suggest that socially isolated and CUMS-treated rats exhibit the manifestation of human depression. The tonifying spleen function of XYS may increase the food consumption and body weight of rats [29]. XYS increases locomotor activity, ameliorates anhedonia, and improves neuroendocrine function and appetite, suggesting that XYS is effective in the prevention and treatment of social isolation and CUMS-induced depression.

In the past decades, evidence has shown the association of MDD with small hippocampal volumes [30, 31]. Structural changes in the volume of the hippocampus [32] and neuronal cell death and abnormal synaptic plasticity in the hippocampus [33] have important functions in the pathophysiology of MDD. Nissl's staining confirmed the injuries of the hippocampal neuronal cell bodies in the model group. The HPA axis is governed by the secretion of CRH from the hypothalamus, and it then stimulates the secretion of CORT from the adrenal cortex [34]. A significant elevation of CRHR2, a high-affinity and membrane-bound receptor for UCN2 [35], was observed in the model group but not CRHR1. CRHR2 signals strongly after binding with urocortin, whereas weak CRHR2 activation occurs only if CRH is released [34]. UCN2 can increase the CORT level in rats treated with the fear-conditioning test [36]. Thus, increased serum UCN 2 and serum CORT were observed in the model group. Selective CRHR2 activation also suppresses the exploration and certain locomotor behavior in rodents [37]. XYS may decrease UCN2 and CRHR2 to ameliorate the depressive-like behavior in rats.

Further proteomic research indicates that PP2A is upregulated in the model group and downregulated by XYS. PP2A is a multimeric protein complex composed of a structural A subunit, a catalytic C subunit, and a variety of targeting B subunits [38]. Previous study shows that social isolation negatively regulates Akt [39] and that the activation of Akt may enhance synaptic plasticity through mTOR [40, 41]. Thus, the phosphorylation of Akt and the expression of mTOR were evaluated. The upregulated phosphorylation of Akt and the expression of mTOR suggest that XYS may improve depressive-like behavior through the PP2A-mediated Akt pathway. Remarkably, *β*-arrestin 2 has an essential function in mediating the interaction of Akt with a targeting B subunit and a catalytic C subunit of PP2A in response to DA [42]. The formation of a protein complex, which is composed of Akt, *β*-arrestin 2, and multimeric protein phosphatase PP2A, is essential for the dephosphorylation and the inactivation of Akt. In vitro and in vivo tests show the antidepressant lithium disruption of the Akt:*β*-arrestin 2:PP2A signaling complex [43, 44]. Thus, the function of *β*-arrestin 2 in social isolation and CUMS-induced depressive-like behavior was assessed.

*β*-arrestin 2 was downregulated in the model group but was improved by XYS and fluoxetine treatment. *β*-arrestin 2 can uncouple CRHR, a G protein-coupled receptor (GPCR), from G proteins and promote its internalization, resulting in desensitization and downregulation [45]. Thus, the decreased expression of CRHR2 might be a result of increased desensitization regulated by *β*-arrestin 2 [46]. *β*-arrestin 2 also functions as a scaffold protein that interacts with several cytoplasmic proteins and links GPCRs to intracellular signaling pathways, such as mitogen-activated protein kinase (MAPK) and Akt [47–49]. *β*-arrestin 2 is a positive mediator of dopaminergic synaptic transmission [42]. The phosphorylation of Akt 308 is upregulated in wild-type mice and downregulated in *β*-arrestin 2 knockout mice upon lithium chloride treatment [43]. Therefore, XYS may increase the expression of *β*-arrestin 2 as a scaffold to enhance the phosphorylation of Akt.

Isolated rats showed a significant decrease in BDNF protein concentrations in the hippocampus [50]. Previous studies also show that phosphorylation of ERK1/2 is downregulated in the hippocampus and prefrontal cortex in rats with depressive-like behavior induced by chronic forced swim stress [51]. Thus, isolation and CUMS might jointly contribute to the downregulation of BDNF in the hippocampus. Fluoxetine alleviates the depressive-like behavior by increasing the phosphorylation of ERK1/2 [52]. The phosphorylation of ERK through *β*-arrestin 2 mediates the src activation, which then potentiates BDNF-stimulated TrkB signaling possibly by trafficking TrkB receptors to neuronal membranes [34, 53]. XYS may increase the phosphorylation of ERK to activate the TrkB pathway, thus alleviating the depressive behavior [54].

In conclusion, social isolation and CUMS induce depressive behavior by upregulating CORT and UCN2. Accordingly, the vicious cycle of HPA hyperactivity deteriorates and causes hippocampal neuron cell body injury. XYS improves the depressive-like behavior through the *β*-arrestin 2 and PP2A-mediated downregulation of CRHR2 and the upregulation of BDNF and mTOR [8]. Further studies will be conducted in vitro to investigate the mechanism of XYS in *β*-arrestin 2-mediated complex formation [42, 44] in regulating GPCR desensitization and scaffolding.

## Supplementary Material

Supplementary Figure S1: Determination of isorhamnetin and ferulic acid in XYS by HPLC-MS/MS.
Supplementary Figure S2: Effects of XYS on body weight and behavior of normal rats.
Supplementary Table S1: Schedule of chronic unpredictable mild stress (CUMS*）*procedure.
Supplementary Table S2: The sequence of primers for qPCR.

## Figures and Tables

**Table 1:** MALDI-TOF MS identification of protein molecules with altered expression.

Number^a^	Accession number^b^	Molecular weight^c^	*p*I^c^	Score^d^	Protein name	Expression level^e^
1	P67779/PHB_RAT	29859	5.57	57 (51)	Prohibitin	↑, ⇑
2	P28073/PSB6_RAT	25502	4.85	39 (26)	Proteasome subunit beta type-6	↑, ⇑
3	P68370/TBA1A_RAT	50788	4.94	64 (25)	Tubulin alpha-1A chain	↓, ⇓
4	N/A^f^					↓, ⇓
5	N/A					↓, ⇓
6	Q4V8B2/DCNL3_RAT	34899	5.07	60 (51)	DCN1-like protein 3	↓, ⇓
7	P36876/2ABA_RAT	52159	5.82	55 (51)	Serine/threonine-protein phosphatase 2A 55 kDa regulatory subunit B alpha isoform	↓, ⇓
8	Q6P9T8/TBB2C_RAT	50225	4.79	83 (26)	Tubulin beta-2C chain	↓, ⇓
9	N/A					↓, ⇓
10	Q8CJ99/SCLT1_RAT	80622	5.98	52 (51)	Sodium channel and clathrin linker 1	↓, ⇓

MALDI-TOF MS: matrix-assisted laser desorption/ionization time of flight mass spectrometer.

^a^defined according to spot positions in 2DE gel indicated, as in Figure 4.

^b^defined from http://www.uniprot.org/.

^c^Molecular weight value calculated by amino acid count; *p*I value calculated from the database entry without any processing.

^d^Protein scores are derived from ions scores as a nonprobabilistic basis for ranking protein hits. Protein scores greater than threshold value are significant (*P* < 0.05).

^e^↑↓ Expression of sham group compared to that of model group; ⇑⇓ expression of XYS group compared to that of model group.

^f^No effective peaks of peptide mass fingerprinting were available.

**Table 2:** Semiquantitative analysis of protein expression and phosphorylation levels by Western blot (WB) and immunohistochemistry (IHC) (mean ± SD).

	Control	Model	XYS	Fluoxetine
BDNF-WB	1.65 ± 0.14	1.32 ± 0.06^▲^	1.49 ± 0.06*	1.56 ± 0.08*
TrkB-WB	1.36 ± 0.08	0.87 ± 0.06^▲^	1.53 ± 0.06^△^	1.37 ± 0.10^#^
p-ERK-WB	1.29 ± 0.05	0.90 ± 0.11^▲^	1.06 ± 0.08^▲∗^	1.18 ± 0.08^#^
ERK-WB	1.15 ± 0.07	1.10 ± 0.05	1.09 ± 0.05	1.13 ± 0.07
*β*-Arrestin 2	2.05 ± 0.11	1.41 ± 0.09^▲^	1.75 ± 0.04^▲#^	1.80 ± 0.04^▲#^
p-mTOR-WB	0.81 ± 0.04	0.28 ± 0.04^▲^	0.76 ± 0.03^#^	0.84 ± 0.03^#^
mTOR-WB	1.22 ± 0.06	1.27 ± 0.05	1.22 ± 0.05	1.23 ± 0.04
p-Akt-WB	0.99 ± 0.05	0.73 ± 0.05^▲^	1.06 ± 0.04^#^	0.95 ± 0.06^#^
Akt-WB	1.00 ± 0.08	1.13 ± 0.08	1.04 ± 0.07	1.09 ± 0.08
PP2A b-WB	1.23 ± 0.06	1.37 ± 0.02^△^	1.13 ± 0.04^#^	1.19 ± 0.10^#^
PP2A c-WB	3.06 ± 0.09	3.16 ± 0.10	3.03 ± 0.07	3.18 ± 0.05
CRHR1-WB	1.54 ± 0.06	1.67 ± 0.14	1.61 ± 0.09	1.70 ± 0.03
CRHR2-WB	0.36 ± 0.02	1.85 ± 0.04^▲^	1.34 ± 0.02^#^	0.42 ± 0.03^△#^
BDNF-IHC	4.07 ± 0.83	1.37 ± 0.52^▲^	4.48 ± 0.89^#^	4.97 ± 1.02^#^
TrkB-IHC	7.52 ± 1.06	1.60 ± 0.54^▲^	5.38 ± 1.13^▲#^	5.95 ± 1.35^#^
p-ERK-IHC	7.07 ± 1.36	2.42 ± 0.79^▲^	5.80 ± 1.25^#^	5.80 ± 1.04^#^
*β*-Arrestin 2-IHC	8.27 ± 1.01	2.77 ± 0.64^▲^	6.58 ± 1.33^△#^	6.72 ± 1.17^△#^
CRHR1-IHC	5.67 ± 1.23	6.25 ± 1.02	5.47 ± 1.26	6.18 ± 1.17
CRHR2-IHC	2.87 ± 0.93	4.27 ± 0.74^▲^	2.58 ± 0.79^#^	2.32 ± 0.63^#^

^△^ *P* < 0.05, ^▲^ *P* < 0.01 versus control, *P < 0.05, ^#^P < 0.01 versus model.
