# Supplementary material for: Xiao Yao San Improves Depressive-Like Behavior in Rats through Modulation of β-Arrestin 2-Mediated Pathways in Hippocampus
Source: Evid Based Complement Alternat Med. 2014 Jul 7;2014:902516. doi: 10.1155/2014/902516 (PMC4109698; doi:10.1155/2014/902516)
Supplement: Supplementary file 1 — Supplementary Figure S1: Determination of isorhamnetin and ferulic acid in XYS by HPLC-MS/MS. Supplementary Figure S2: Effects of XYS on body weight and behavior of normal rats. Supplementary Table S1: Schedule of chronic unpredictable mild stress (CUMS）procedure. Supplementary Table S2: The sequence of primers for qPCR. [file 902516.f1.pdf]

Supplementary materials

1. Sample preparation

Blood was collected from the abdominal aorta using a puncturing needle at 1 h and 6 h after intragastric administration of Xiao Yao San (XYS). The supernatant was placed in room temperature for 30 min, centrifuged for 10 min at 3000 × *g*, and then extracted. The serum was stored at -80 °C.

About 200 μl of the serum and 400 μl of acetonitrile were mixed and vortexed for 30 s. After centrifugation at 13,000 rpm for 10 min at 4 °C, the supernatants were loaded to the high-performance liquid chromatography-mass spectrometry (HPLC-MS) for fingerprint analysis.

HPLC-MS conditions

HPLC-MS/MS analysis was performed with an API 4000-QTRAP® LC/MS/MS System (AB SCIEX, Framingham, MA, USA). A Zorbax Eclipse C_{18} column (50 × 2.1 mm, i.d. 3.5 μm, Agilent, USA) was used for chromatographic separations. Column temperature was maintained at 40 °C. The samples were separated using a gradient mobile phase consisting of CHOH (A) and H_2O-HCOOH (B) (100:0.1,*v*/*v*). The flow rate was 0.3 ml/min. About 10 μl of the sample solution was injected in each run. HPLC effluent was introduced directly to the electrospray source operated in a positive ionization mode and connected to a triple quadrupole mass spectrometer.

The compound was ionized in the electrospray ionization operated in the positive mode. Ionizing voltage was 5000 V, and ion source temperature was 600 °C. Curtain gas: 30, GS1: 60, GS2: 60. Total ion current chromatograms were obtained by a mass spectrometer in multiple monitoring modes. The ion pairs used for the qualitative analysis were m/z 315.2 → m/z 300.3 and m/z 315.2 → m/z 151.2 (isorhamnetin); m/z 193.6 → m/z 135.6 and m/z 193.6 → m/z 150.5 (ferulic acid); m/z 353.8 → m/z 191.7 and m/z 353.8 → m/z 86.0 (chlorogenic acid); and m/z 783.5 → m/z 622.5 and m/z 783.5 → m/z 652.4 (astragaloside).

Software Analyst®1.5 was used for controlling the instruments and data collection and processing.

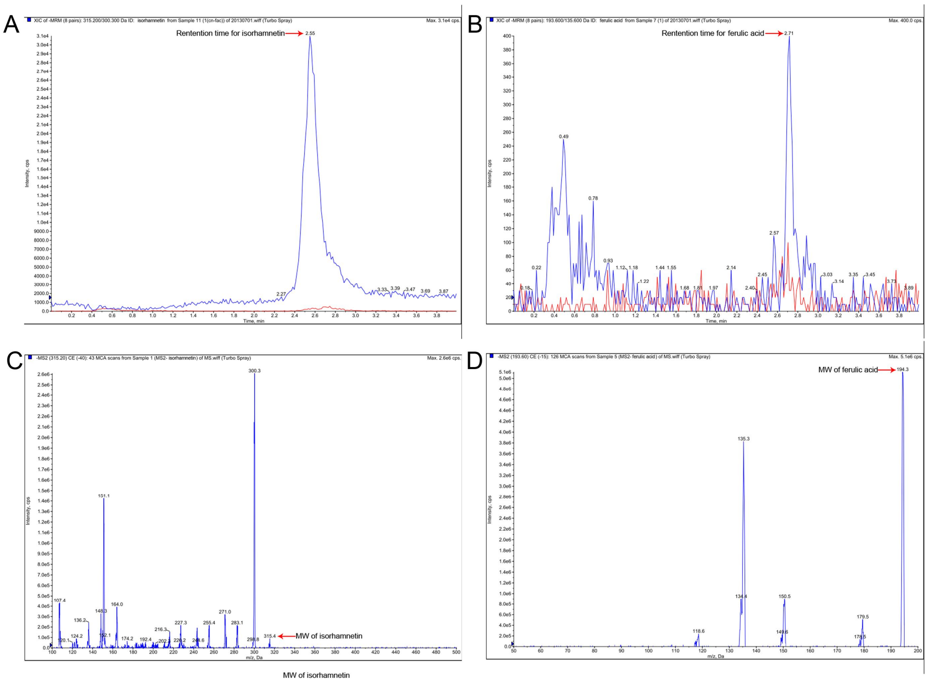

Figure S1. Determination of isorhamnetin and ferulic acid in XYS by HPLC-MS/MS.

Serum sample from a rat 1 h after intragastric administration of 1.9 $mg \cdot kg^{-1}$ XYS. The retention time for isorhamnetin (A) by HPLC MS/MS was 2.55 min. Serum sample was from a rat 6 h after XYS treatment. The retention time for ferulic acid (B) was 2.71 min. The corresponding molecular weights of isorhamnetin (C) and ferulic acid (D) were determined by HPLC MS/MS.

2. Animals and experimental procedures

A total of 20 male Sprague-Dawley rats, weighing 200 ± 20g, were purchased from the Center of Experimental Animals, Southern Medical University. The animals were maintained under controlled conditions (22 °C, 12 h/12 h dark/light cycle) in a conventional animal colony for 3 days to adapt to the new environment.

Rats were assigned randomly into two groups: Control and Control+XYS. Five animels per cage were housed and allowed free access to food and water. About 19g/Kg/d XYS (for Control+XYS group) and an equivalent volume of distilled water (for Control group) were administrated by gavage using a tube twice a day.

We did Behavior tests at day 0 and day 21, and measured their body weight on the last day of the week. The results were shown in figure S2.

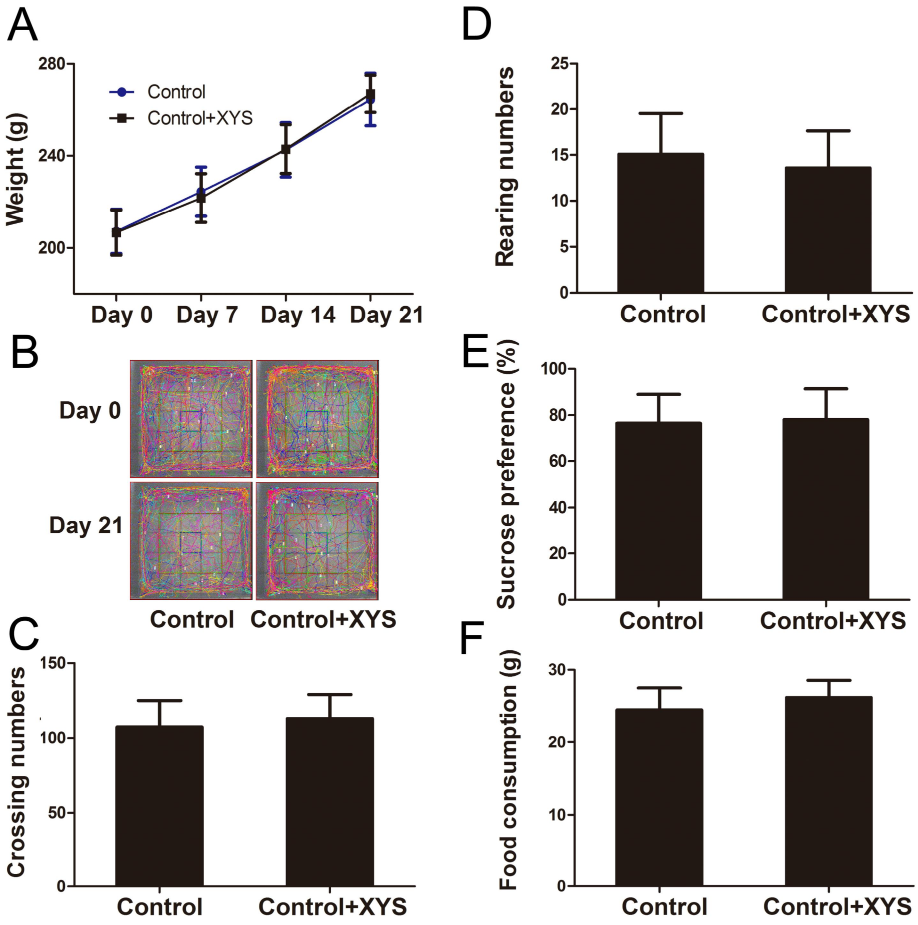

Figure S2. Effects of XYS on body weight and behavior of normal rats. Body weight was measured once a week (A). A battery of behavioral tests was initiated 21 d after intragastric administration of XYS, and the following parameters were measured: crossing trajectories (B), crossing numbers (C), rearing numbers (D), sucrose preference (E), and food consumption (F). Data are expressed as mean ± SD, n = 10 per group. No significant difference was found between the two groups on body weight and behavior.

Table S1 Schedule of chronic unpredictable mild stress（CUMS）procedure

Day	Food deprivation	Water deprivation	Empty bottle	Cage title	Overnight illumination	Soiled cage	Forced swimming	Restraint	Foreign object
Monday	9:30	9:30							9:30
	↓	↓							↓
Tuesday	9:30	9:30	9:30						9:30
			↓						
Wednesday			10:30				9:30		
							↓		
					19:00		10:00		
					↓				
Thursday					7:00			9:30	
								↓	
				12:00				12:30	
				↓					
				19:00					
Friday	9:30					9:30			
	↓					↓			
Saturday	9:30	10:00				9:30			
		↓			19:00				
					↓				
Sunday		10:00	10:00		7:00				
			↓						
			11:00	11:00					
				↓					
				18:00					

Table S2 The sequence of primers for qPCR

Primer Name		Primer Sequences
PP2A b	F	TGTTGTTGGAATGGGTCTGA
	R	CAGACTTTGCGTGGTTTCAA
PP2A c	F	CTCTCACTGCCTTGGTGGAT
	R	TGACCACAGCAAGTCACACA

GAPDH	F	ATTGTCAGCAATGCA TCCTG
	R	ATGGACTGTGGTCATGAGCC
